# Evaluating reproducibility of AI algorithms in digital pathology with DAPPER

**DOI:** 10.1371/journal.pcbi.1006269

**Published:** 2019-03-27

**Authors:** Andrea Bizzego, Nicole Bussola, Marco Chierici, Valerio Maggio, Margherita Francescatto, Luca Cima, Marco Cristoforetti, Giuseppe Jurman, Cesare Furlanello

**Affiliations:** 1 Fondazione Bruno Kessler, Trento, Italy; 2 DIPSCO, University of Trento, Trento, Italy; 3 Department CIBIO, University of Trento, Trento, Italy; 4 Pathology Unit, Santa Chiara Hospital, Trento, Italy; University of Virginia, UNITED STATES

## Abstract

Artificial Intelligence is exponentially increasing its impact on healthcare. As deep learning is mastering computer vision tasks, its application to digital pathology is natural, with the promise of aiding in routine reporting and standardizing results across trials. Deep learning features inferred from digital pathology scans can improve validity and robustness of current clinico-pathological features, up to identifying novel histological patterns, *e.g*., from tumor infiltrating lymphocytes. In this study, we examine the issue of evaluating accuracy of predictive models from deep learning features in digital pathology, as an hallmark of reproducibility. We introduce the DAPPER framework for validation based on a rigorous Data Analysis Plan derived from the FDA’s MAQC project, designed to analyze causes of variability in predictive biomarkers. We apply the framework on models that identify tissue of origin on 787 Whole Slide Images from the Genotype-Tissue Expression (GTEx) project. We test three different deep learning architectures (VGG, ResNet, Inception) as feature extractors and three classifiers (a fully connected multilayer, Support Vector Machine and Random Forests) and work with four datasets (5, 10, 20 or 30 classes), for a total of 53, 000 tiles at 512 × 512 resolution. We analyze accuracy and feature stability of the machine learning classifiers, also demonstrating the need for diagnostic tests (*e.g*., random labels) to identify selection bias and risks for reproducibility. Further, we use the deep features from the VGG model from GTEx on the KIMIA24 dataset for identification of slide of origin (24 classes) to train a classifier on 1, 060 annotated tiles and validated on 265 unseen ones. The DAPPER software, including its deep learning pipeline and the Histological Imaging—Newsy Tiles (HINT) benchmark dataset derived from GTEx, is released as a basis for standardization and validation initiatives in AI for digital pathology.

## Author summary

In this study, we examine the issue of evaluating accuracy of predictive models from deep learning features in digital pathology, as an hallmark of reproducibility. It is indeed a top priority that reproducibility-by-design gets adopted as standard practice in building and validating AI methods in the healthcare domain. Here we introduce DAPPER, a first framework to evaluate deep features and classifiers in digital pathology, based on a rigorous data analysis plan originally developed in the FDA’s MAQC initiative for predictive biomarkers from massive omics data. We apply DAPPER on models trained to identify tissue of origin from the HINT benchmark dataset of 53, 000 tiles from 787 Whole Slide Images in the Genotype-Tissue Expression (GTEx) project, available at the web address https://gtexportal.org. We analyze accuracy and feature stability of different deep learning architectures (VGG, ResNet and Inception) as feature extractors and classifiers (a fully connected multilayer, Support Vector Machine and Random Forests) on up to 20 classes. Further, we use the deep features from the VGG model (trained on HINT) on the 1, 300 annotated tiles of the KIMIA24 dataset for identification of slide of origin (24 classes). The DAPPER software is available together with the scripts to generate the HINT benchmark dataset.

## Introduction

Artificial Intelligence (AI) methods for health data hold great promise but still have to deal with disease complexity: patient cohorts are most frequently an heterogeneous group of subtypes diverse for disease trajectories, with highly variable characteristics in terms of phenotypes (*e.g*. bioimages in radiology or pathology), response to therapy, clinical course, thus a challenge for machine-learning based prognoses. Nevertheless, the increased availability of massive annotated medical data from health systems and a rapid progress of machine learning frameworks has led to high expectations about the impact of AI on challenging biomedical problems [1]. In particular, Deep Learning (DL) is now surpassing pattern recognition methods in the most complex medical images challenges such as those proposed by the Medical Image Computing & Computer Assisted Intervention conferences (MICCAI, https://www.miccai2018.org/en/WORKSHOP---CHALLENGE---TUTORIAL.html), and it is comparable to expert accuracy in the diagnosis of skin lesions [2], classification of colon polyps [3, 4], ophthalmology [5], radiomics [6] and other areas [7]. However, the reliable comparison of DL with other baseline ML models and human experts is not a diffuse practice yet [8], and also the reproducibility and interpretation of the challenges’ outcome have been recently criticized [9, 10]. DL refers to a class of machine learning methods that model high-level abstractions in data through the use of modular architectures, typically composed by multiple nonlinear transformations estimated by training procedures. Notably, deep learning architectures based on Convolutional Neural Networks (CNNs) hold state-of-the-art accuracy in numerous image classification tasks without prior feature selection. Further, intermediate steps in the pipeline of transformations implemented by CNNs or other deep learning architectures can provide a mapping (*embedding*) from the original feature space into a *deep feature* space. Of interest for medical diagnosis, deep features can be used for interpretation of the model and can be directly employed as inputs to other machine learning models.

Deep learning methods have been applied to analysis of histological images for diagnosis and prognosis. Mobadersany and colleagues [11] combine in the Survival Convolutional Neural Network (SCNN) architecture a CNN with traditional survival models to learn survival-related patterns from histology images, predicting overall survival of patients diagnosed with gliomas. Predictive accuracy of SCNN is comparable with manual histologic grading by neuropathologists. Further, by incorporation of genomic variables for gliomas in the model, the extended model significantly outperforms the WHO paradigm based on genomic subtype and histologic grading. Similarly, deep learning models have been successfully applied to histology for colorectal cancer [12], gastric cancer [13], breast cancer [14] and lung cancer [15, 16].

As human assessments of histology are subjective and hard to repeat, computational analysis of histology imaging within the information environment generated from a digital slide (*digital pathology*) and advances in scanning microscopes have already allowed pathologists to gain a much more effective diagnosis capability and dramatically reduce time for information sharing. Starting from the principle that underlying differences in the molecular expressions of the disease may manifest as tissue architecture and nuclear morphological alterations [17], it is clear that automatic evaluation of disease aggressiveness level and patient subtyping has a key role aiding therapy in cancer and other diseases. Digital pathology is in particular a key tool for the immunotherapy approach, which stands on the characterization of tumor-infiltrating lymphocytes (TILs) [18]. Indeed, quantitative analysis of the immune microenvironment by histology is crucial for personalized treatment of cancer [19, 20], with high clinical utility of TILs assessment for risk prediction models, adjuvant, and neoadjuvant chemotherapy decisions, and for developing the potential of immunotherapy [21, 22]. Digital pathology is thus a natural application domain for machine learning, with the promise of accelerating routine reporting and standardizing results across trials. Notably, deep learning features learned from digital pathology scans can improve validity and robustness of current clinico-pathological features, up to identifying novel histological patterns, *e.g*. from TILs.

On the technical side, usually deep learning models for digital pathology are built upon imaging architectures originally aimed at tasks in other domains and trained on non-medical datasets. This is a foundational approach in machine learning, known as *transfer learning*. Given domain data and a network pretrained to classify on huge generic databases (*e.g*. ImageNet, with over 14 million items and 20 thousand categories [23]), there are three basic options for transfer learning, *i.e*. to adapt the classifier to the new domain: a) train a new machine learning model on the features preprocessed by the pretrained network from the domain data; b) retrain only the deeper final layers (the *domain layers*) of the pretrained network; c) retrain the whole network starting from the pretrained state. A consensus about the best strategy to use for medical images is still missing [24, 25].

In this study we aim to address the issue of reproducibility and validation of machine learning models for digital pathology. Reproducibility is a paramount concern in biomarker research [26], and in science in general [27], with scientific communities, institutions, industry, and publishers struggling to foster adoption of best practices, with initiatives ranging from enhancing reproducibility of high-throughput technologies [28] to improving the overall reuse of scholarly data and analytics solutions (*e.g*. the FAIR Data Principles [29]). As an example, the MAQC initiatives [30, 31], led by the US FDA, investigate best practices and causes of variability in the development of biomarkers and predictive classifiers from massive omics data (*e.g*. microarrays, RNA-Seq or DNA-Seq data) for precision medicine. The MAQC projects adopt a Data Analysis Plan (DAP) that forces bioinformatics teams to submit classification models, top features ranked for importance and performance estimates all built on training data only, before testing on unseen external validation data. The DAP approach is methodologically more robust than a simple cross validation (CV) [30] as the internal CV and model selection phase is replicated multiple times (*e.g*., 10 times) to smooth the impact of a single training/test split; the performance metrics is thus evaluated on a much larger statistics. Also, features are analyzed and ranked multiple times, averaging the impact of a small round of partitions. The ranked feature lists are fused in a single ranked list using the Borda method [32] and the bootstrap method is applied to compute the confidence intervals. This approach helps mitigating the risk of selection bias in complex learning pipelines [33], where the bias can stem in one of many preprocessing steps as well as in the downstream machine learning model. Further, it clarifies that increasing task difficulty is often linked to a decrease not only in accuracy measures but also of stability of the biomarker lists [32], *i.e*. the consistency in the selection of the top discriminating features across all repeated cross validation runs.

Although openness in sharing algorithms and benchmark data is a solid attitude of the machine learning community, the reliable estimation on a given training dataset of predictive accuracy and stability of deep learning models (in terms of performance range as a function of variations of training data) and the stability of deep features used by external models (as the limited difference of top ranking variables selected by different models) is still a gray area. The underlying risk is that of overfitting the training data, or worse to overfit the validation data if the labels are visible, which is typical when datasets are fully released at the end of a data science challenge on medical image data. As the number of DL-based studies in digital pathology is exponentially growing, we suggest that the progress of this field needs environments (*e.g*., DAPs) to prevent such pitfalls, especially if features distilled by the network are used as radiomics biomarkers to inform medical decision. Further, given an appropriate DAP, alternative model choices should be benchmarked on publicly available datasets, as usual in the general computer vision domain (*e.g*., ImageNet [23] or COCO [34]).

This study provides three main practical contributions to controlling for algorithmic bias and improving reproducibility of machine learning algorithms for digital pathology:

1. A Data Analysis Plan (DAP) specialized for digital pathology, tuned on the predictive evaluation of deep features, extracted by a network and used by alternative classification heads. To the best of our knowledge, this is the first study where a robust model validation method (the DAP) is applied in combination with the deep learning approach. We highlight that the approach can be adopted in other medical/biology domains in which Artificial Intelligence is rapidly emerging, *e.g*., in the analysis of radiological images.
2. A benchmark dataset (HINT) of 53, 727 tiles of histological images from 30 tissue types, derived from GTEx [35] for the recognition of tissue of origin of up to 30 classes. The HINT dataset can be used by other researchers to pretrain the weights of DL architectures that shall be applied on digital pathology tasks (*e.g*., detection of TILs) thus accelerating the training of application-specific models. In the past 5 years, having a shared image dataset (*e.g*., the ImageNet) allowed the development of a number of deep learning models for general image classification (*e.g*. VGG, ResNet, AlexNet). Such pretrained networks have then been effectively applied on a variety of different tasks. With the HINT dataset we aim at favouring a similar boost on digital pathology.
3. An end-to-end machine learning framework (DAPPER) as a baseline environment for predictive models in digital pathology, where end-to-end indicates that the DAPPER framework is directly applied to the digital pathology images, with the deep learning component producing features for the machine learning head, without an external procedure (*e.g*., a handcrafted feature extraction) to preprocess the features. To the best of our knowledge, this is the first example of a DL approach for the classification of up to 30 different tissues, all with the same staining, which represents, *per se*, a valuable contribution to the digital pathology community.

**Fig 1:**
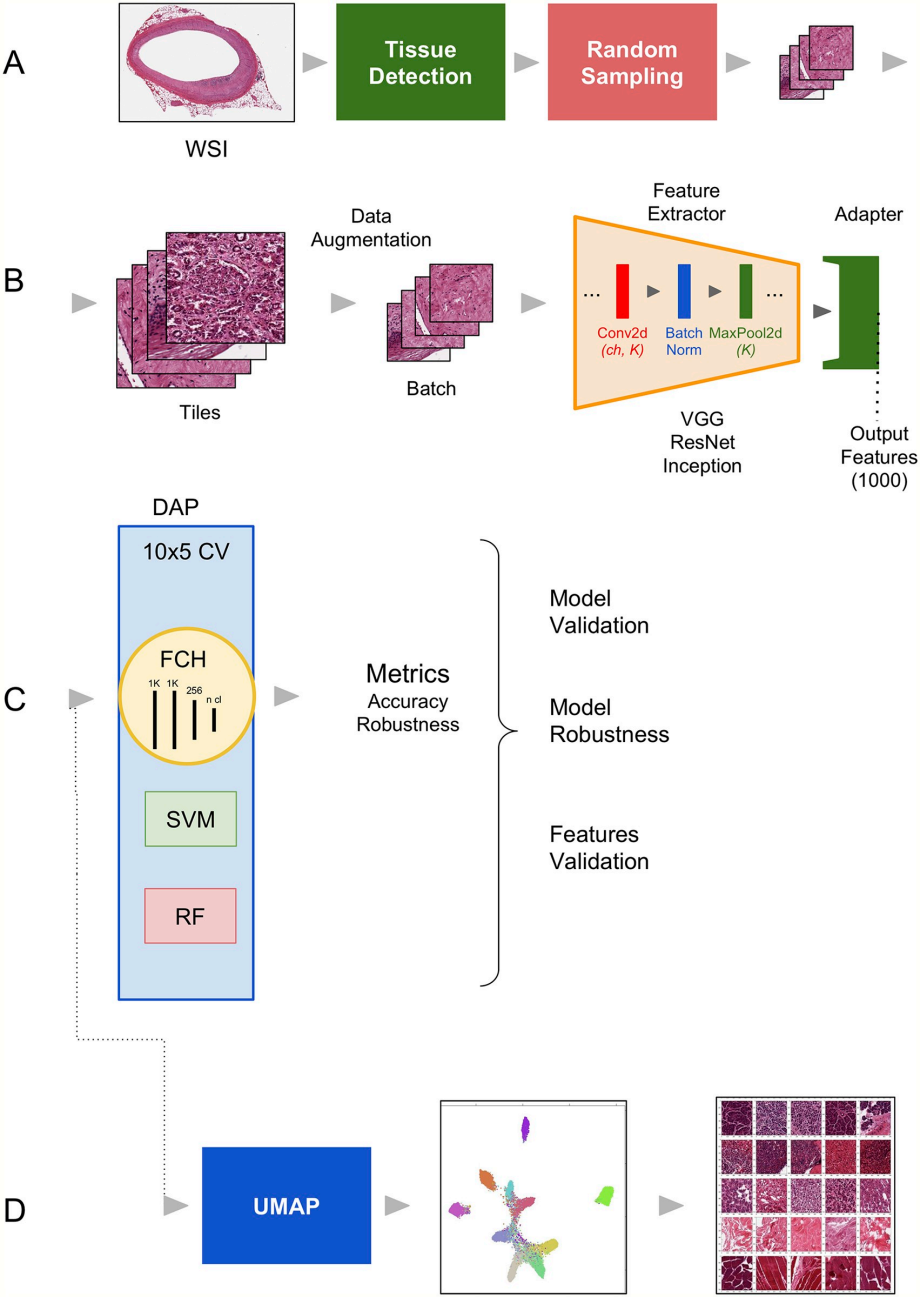
The DAPPER environment. Components: A) The WSI preprocessing pipeline; B) the deep learning backend to extract deep features; C) the Data Analysis Plan (DAP) for the machine learning models; and D) the UMAP module and other modules for unsupervised analysis.

We first apply DAPPER to a set of classification experiments on 787 Whole Slide Images (WSIs) from GTEx. The framework (see Fig 1) is composed by (A) a preprocessing component to derive patches from WSIs; (B) a 3-step machine learning pipeline with a data augmentation preprocessor, a backend deep learning model, and an adapter extracting the deep features; (C) a downstream machine learning/deep learning head, *i.e*. the task specific predictor. In our experiments, we evaluate the accuracy and the feature stability in a multiclass setting for the combination of three different deep learning architectures, namely VGG, ResNet and Inception, used as feature extractors, and three classifiers, a fully connected multilayer network, Support Vector Machine (SVM) [36] and Random Forest (RF) [37]. This component is endowed with the DAP, *i.e*., a 10 × 5 CV (5-fold cross validation iterated 10 times). The 50 internal validation sets are used to estimate a vector of metrics (with confidence intervals) that are then used for model selection. In the fourth component (D) we finally provide an unsupervised data analysis based on the UMAP projection method, and methods for feature exploration. The DAPPER software is available together with the Python scripts and the instructions to generate the HINT benchmark dataset as a collection of Jupyter notebooks at gitlab.fbk.eu/mpba-histology/dapper, released under the GNU General Public License v3. Notably, the DAP estimates are provided in this paper only for the downstream machine learning/deep learning head in component (C); whenever computational resources are available, the DAP can be expanded also to component (B). Here we kept as a separate problem the model selection exercise on the backend deep learning architecture in order to clarify the change of perspective with respect to optimization of machine learning models in the usual training-validation setting.

As a second experiment, in order to study the DAPPER framework in a transfer learning condition, we use the deep features from the VGG model trained on a subset of HINT on the 1, 300 annotated tiles of the KIMIA Path24 dataset [38] to identify in this case the slide of origin (24 classes).

Previous work on classifying WSIs by means of neural networks was introduced by [38, 39], also with the purpose of distributing the two original datasets KIMIA Path960 (KIMIA960) and KIMIA Path24 (KIMIA24). KIMIA24 consists of 24 WSIs chosen on purely visual distinctions. Babaie and coauthors [38] manually selected a total of 1, 325 binary patches with 40% overlap. On this dataset, in addition to two models based on Local Binary Patterns (LBP) and Bag-of-Visual-Words (BoVW), they applied two shallow CNNs, achieving at most 41.8% accuracy. On the other hand, KIMIA960 contains 960 histopathological images belonging to 20 different WSIs that, again on visual clues, were used to represent different texture/pattern/staining types. The very same experimental settings as the one for KIMIA24, *i.e*., LBP, BoVW and CNN, has been replicated on this dataset by Kumar and coauthors [39]. In particular, the authors applied AlexNet or VGG16, both pretrained on ImageNet, to extract deep features; instead of a classifier, accuracy was established by computing similarity distances between the 4, 096 features extracted. Also, Kieffer and coauthors in [25] explored the use of deep features from several pretrained structures on KIMIA24, controlling for the impact of transfer learning and finding an advantage of pretrained networks against training from scratch. Conversely, Alhindi and coworkers [40] analyzed KIMIA960 for slide of origin (20 slides preselected by visual inspection), and similarly to our study they compared alternative classifiers as well as feature extraction models in a 3-fold CV setup. Considering the importance of clinical validation of predictive results [8], we finally compared the performance of the DAPPER framework with an expert pathologist. DAPPER outperforms the pathologist in classifying tissues at tile level, while at WSI level performance are similar.

DAPPER represents an advancement over previous studies, due to the DAP structure and its application to the large HINT dataset free of any visual preselection.

## Materials and methods

### Dataset

The images used to train the models were derived from the Genotype-Tissue Expression (GTEx) Study [35]. The study collects gene expression profiles and whole-slide images (WSIs) of 53 human tissues histologies used to investigate the relationship between genetic variation and tissue-specific gene expression in healthy individuals. To ensure that the collected tissues meet prescribed standard criteria, a Pathology Resource Center validated each sample origin, content, integrity and target tissue (https://biospecimens.cancer.gov/resources/sops/). After sectioning and Haemotoxylin and Eosin staining (H&E), tissue samples were scanned using a digital whole slide imaging system (Aperio) and stored in .*svs* format [41].

A custom Python script was used to download 787 WSIs through the Biospecimen Research Database (total size: 192 GB, average 22 WSIs for each tissue). The list of the downloaded WSIs is available in S1 Table.

**Fig 2:**
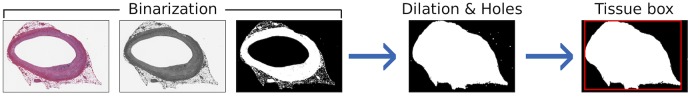
The tissue detection pipeline. The identification of the tissue bounding box is performed on the WSI thumbnail in three steps: a) Binarization of the grayscale image by applying Otsu’s thresholding; b) Binary dilation and filling of the holes; c) Selection of the biggest connected region as tissue region and computation of the vertex of the containing rectangle.

A data preprocessing pipeline was developed to prepare the WSIs as training data (see Fig 2). The WSIs have a resolution of 0.275 *μ*m/pixel (Magnification 40*X*) and variable dimensions. Further, the region interested by the tissue is only a portion of the WSI and it varies across the samples. Hence first we identified the region of the tissue in the image (see Fig 2), then we extracted at most 100 tiles (512 × 512 pixel) from the WSIs, by randomly sampling the tissue region. We applied the algorithm for the detection of the tissue region (see Fig 2) on each tile and rejected those where the portion of the tissue was below 85%. A total number of 53, 727 tiles was extracted, with a number of tiles per tissue varying between 59 (for Adipose—Visceral (Omentum)) and 2, 689 (for Heart—Left Ventricle). Four datasets (HINT5, HINT10, HINT20, HINT30) have been derived with increasing number of tissues for a total of 52, 991 tiles; the full number of tiles per anatomical zone, for each dataset, is available in S2 Table and summarized in Table 1. We refer to the four sets as the HINT collection, or the HINT dataset in brief. We choose the five tissues composing HINT5 based on exploratory experiments, while the other three datasets were composed including the tissues with higher number of tiles. The class imbalance is accounted for by weighting the error on predictions. In detail, the weight *w* of the class *i* used in the cross entropy function is computed as: *w*_*i*_ = *n*_max_/*n*_*i*_, where *n*_max_ is the number of tiles in the class with more tiles and *n*_*i*_ is the number of tiles in the class *i*.

**Table 1:** Summary of the HINT datasets. Total: total number of tiles composing the dataset; Min: number of tiles in the class with less samples; Max: number of tiles in the class with more samples; Average; average number of tiles for each class.

Name	# tissues	Total	Min	Max	Average
HINT5	5	8, 218	1, 009	2, 424	1, 643.6
HINT10	10	22, 885	1, 890	2, 689	2, 288.5
HINT20	20	40, 516	1, 574	2, 689	2, 025.8
HINT30	30	52, 991	957	2, 689	1, 766.4

Since image orientation should not be relevant for the tissue recognition, the tiles are randomly flipped (horizontally and vertically) and scaled, following a common practice in deep learning known as *data augmentation*. Data augmentation consists of different techniques (such as cropping, flipping, rotating images) performed each time a sample is loaded, so that the resulting input image is different at each epoch. Augmentation has proven effective in multiple problems, increasing the generalization capabilities of the network, preventing overfitting and improving models performance [42–44].

Such randomized transformations were found to provide more comparable performance between the prognostic accuracy of the deep learning SCNN architecture and that of standard models (*i.e*., Support Vector Machine, Random Forest) based on combined molecular subtype and histologic grade [11]. In addition, each tile is cropped to a fixed size, which is dependent on the type of network used to extract the deep features.

### Deep learning architectures and training strategies

We exploited three backend architectures commonly used in computer vision tasks:

1. VGG, Net-E version (19 layers) with Batch Normalization (BN) layers [45];
2. ResNet, 152-layer model [46];
3. Inception, version 3 [47].

These architectures have reached highest accuracy in multiclass classification problems over the last 4 years [48] and differ in resource utilization (see Table 2). The feature extraction layer of each backend network is obtained as the output of an end-to-end pipeline composed of the following main blocks (see panel B in Fig 1):

1. Data augmentation: the input tiles are processed and assembled into batches of size 32;
2. Feature Extractor: series of convolutional layers (Conv2d: with different number of channels and kernel size), normalization layers (Batch Norm) and pooling layers (MaxPool2d: with different kernel size) designed to fit with the considered backend architecture (VGG, ResNet, Inception). The number of output features of the Feature Extractor depends on the structure of the backend architecture used;
3. Adapter: as the backend networks have output features of different sizes, we add a linear layer at the end of the Feature Extractor, in order to make the pipeline uniform. The Adapter takes the features of the backend network as input and output a fixed number of features (1, 000).

**Table 2:** Backend architectures statistics.

Name	Output features	#Parameters	Layers
VGG	25, 088	155 × 10^6^	19
ResNet	2, 048	95 × 10^6^	152
Inception	2, 048	35 × 10^6^	42

The 1, 000 Adapter features are then used as input for a classifier providing predicted tissue labels as output. As predictive models, we used a linear SVM with regularization parameter *C* set to 1, a RF classifier with 500 trees (both implemented in *scikit-learn*, v0.19.1) and a fully connected head (FCH), namely a series of fully connected layers (see panel C in Fig 1). Inspired by [11] and [49], our FCH consists of four dense layers with 1, 000, 1, 000, 256 and *number of tissue classes* nodes, respectively. The feature extraction block was initialized with the weights already trained on the ImageNet dataset [23], provided by *PyTorch* (v0.4.0) and frozen. The Adapter block is trained together with the FCH as a one network. Training also the weights of the feature extraction block improves accuracy (see S3 Table). However, these results were not validated rigorously within the DAP and therefore they not are not claimed as generalized in this study.

For the optimization of the other weights (Adapter and FCH) we used the Adam algorithm [50] with the learning rate set to 10^−5^ and fixed for the whole training. We used the cross entropy as the loss function, which is appropriate for multiclass models.

The strategy to optimize the learning rate was selected based on results of a preparatory study with the VGG network and HINT5. The strategy approach with fixed learning rate achieved the best results (see S4 Table) and was therefore adopted in the rest of the study.

### Data analysis plan

Following the rigorous model validation techniques proposed by the MAQC projects [30, 31], we adopted a DAP to assess the validity of the features extracted by the networks, namely a 10 × 5-fold cross validation (CV) schema. The input dataset is first partitioned in two separate datasets, the *training set* and the *test set*, also referred as *external validation set* as reported in [30, 31]. The external validation set will be kept completely unseen to the model, and it will be only used in the very last step of the DAP for the final model evaluation. In our experimental settings, we used 80% of the total samples for the training set, and the remaining 20% for the external validation set. A stratification strategy upon the classes of tiles, *i.e*., 5, 10, or 20, has been adopted in the partitioning. The training set further undergoes a 5-fold CV iterated 10 times, resulting in 50 separated *internal validation sets* used for model evaluation within the DAP. The same stratification strategy is used in the creation of the folds.

At each CV iteration, features are ranked by KBest, with ANOVA F-score as the scoring function [51], and four separate models are trained on sets of increasing number of ranked features (namely: 10%, 25%, 50%, 100% of the total number of features). A list of top-ranked features is obtained by Borda aggregation of the ranked lists, for which we also compute the Canberra stability with a computational framework designed for sets of ranked biomarker lists [32].

As for model evaluation, we considered the accuracy (ACC), and the Matthews Correlation Coefficient (MCC) in their multiclass generalization [52–54]:

$$\begin{array}{c} \text{ACC}=\frac{\sum_{k=1}^{N}C_{kk}}{\sum_{i,j=1}^{N}C_{ij}},\;0\leq \text{ACC}\leq 1 \end{array}$$ (1)

$$\begin{array}{c} \text{MCC}=\frac{\sum_{k,l,m=1}^{N}\left(C_{kk}C_{ml}-C_{lk}C_{km}\right)}{\sqrt{\sum_{k=1}^{N}[\sum_{l=1}^{N}C_{lk}\sum_{\begin{array}{c} f,g=1\\ f\neq k \end{array}}^{N}C_{gf}]}\sqrt{\sum_{k=1}^{N}[\sum_{l=1}^{N}C_{kl}\sum_{\begin{array}{c} f,g=1\\ f\neq k \end{array}}^{N}C_{fg}]}},\;-1\leq \text{MCC}\leq 1 \end{array}$$ (2)

where *N* is the number of classes and *C*_*st*_ is the number of elements of true class *s* that have been predicted as class *t*.

MCC is widely used in Machine Learning as a performance metric, especially for unbalanced sets, for which ACC can be misleading [55]. In particular, MCC gives an indication of prediction robustness among classes: MCC = 1 is perfect classification, MCC = −1 is extreme misclassification, and MCC = 0 corresponds to random prediction.

Finally, the overall performance of the model is evaluated across all the iterations (*i.e*., internal validation sets), in terms of average MCC and ACC with 95% Studentized bootstrap confidence intervals (CI) [56], and then on the external validation set.

As a sanity check to avoid unwanted selection bias effects, the DAP is repeated stochastically scrambling the training set labels (*random labels* mode) or by randomly ranking features before building models (*random ranking* mode: in presence of pools of highly correlated variables, top features can be interchanged with others, possibly of higher biological interest). In both modes, a procedure unaffected by selection bias should achieve an average MCC close to 0.

### Experiments on HINT

We designed a set of experiments reported in Table 3 to provide indications about the optimal architecture for deep feature extraction, while keeping fixed the other hyper-parameters. In particular we set batch size (32) and number of epochs (50), large enough to let the network converge: we explored increasing numbers of epochs (10, 30, 50, 100) and, since the loss stabilizes after about 35 epochs, we set the number of epochs to 50. First, we compared the three backend architectures on the smallest dataset HINT5, with fixed learning rate. Both VGG and ResNet architectures achieved good results, outperforming Inception as shown in Tables 4 and 5. In successive analyses we thus restricted to use VGG and ResNet as feature extractors and validated performance and features with the DAP. The same process was adopted on HINT10 and HINT20. An experiment with 30 tissues has also been performed. Results are listed in S5 Table.

**Table 3:** Summary of experiments with the backend architectures.

Experiment	Dataset	Feature extractor	Version/Model
VGG-5	HINT5	VGG	Net-E+BN
ResNet-5	HINT5	ResNet	152-layer
Inception-5	HINT5	Inception	3
VGG-10	HINT10	VGG	Net-E+BN
ResNet-10	HINT10	ResNet	152-layer
VGG-20	HINT20	VGG	Net-E+BN
ResNet-20	HINT20	ResNet	152-layer

**Table 4:** Matthew correlation coefficient values for each experiment, and classifier head pairs on HINT dataset. The average cross validation MCC with 95% CI (**H-MCCt**), and MCC on the external validation set (**H-MCCv**) are reported. Best-performing backend network, and classifier head combination on each dataset are reported in bold.

	FCH		SVM		RF	
Experiment	H-MCCt	H-MCCv	H-MCCt	H-MCCv	H-MCCt	H-MCCv
VGG-5	0.841 (0.838, 0.843)	0.820	0.786 (0.783, 0.789)	0.777	0.750 (0.748, 0.753)	0.747
ResNet-5	**0.879 (0.877, 0.881)**	**0.883**	0.852 (0.850, 0.854)	0.840	0.829 (0.827, 0.832)	0.849
Inception-5			0.747 (0.744, 0.750)	0.734	0.703 (0.699, 0.706)	0.701
VGG-10	**0.896 (0.894, 0.897)**	**0.894**	0.861 (0.859, 0.862)	0.866	0.889 (0.888, 0.891)	0.886
ResNet-10	0.857 (0.856, 0.859)	0.860	0.825 (0.824, 0.827)	0.832	0.845 (0.843, 0.847)	0.850
VGG-20	0.771 (0.770, 0.772)	0.774	0.729 (0.727, 0.730)	0.731	0.761 (0.760, 0.762)	0.766
ResNet-20	0.756 (0.754, 0.757)	0.757	**0.788 (0.787, 0.789)**	**0.792**	0.738 (0.737, 0.739)	0.738

**Table 5:** Accuracy values for each experiment, and classifier head pairs on HINT dataset. The average cross validation ACC with 95% CI and ACC on the external validation set are reported. Best-performing backend network, and classifier head combination on each dataset are reported in bold.

	FCH		SVM		RF	
Experiment	H-ACCt	H-ACCv	H-ACCt	H-ACCv	H-ACCt	H-ACCv
VGG-5	87.2 (87.0, 87.5)	85.6	82.9 (82.7, 83.1)	82.1	79.9 (79.7, 80.1)	79.7
ResNet-5	**90.3 (90.1, 90.5)**	**90.7**	88.1 (88.0, 88.3)	87.2	86.3 (86.1, 86.5)	87.9
Inception-5			79.8 (79.5, 80.0)	78.7	76.2 (75.9, 76.4)	75.9
VGG-10	**90.6 (90.5, 90.7)**	**90.5**	87.5 (87.3, 87.6)	88.0	90.0 (89.9, 90.2)	89.7
ResNet-10	87.2 (87.0, 87.3)	87.4	84.3 (84.1, 84.4)	84.9	86.1 (85.9, 86.2)	86.5
VGG-20	78.2 (78.1, 78.4)	78.5	74.1 (74.0, 74.2)	74.4	77.3 (77.2, 77.4)	77.7
ResNet-20	76.7 (76.6, 76.9)	76.9	**79.9 (79.8, 80.0)**	**80.3**	75.1 (75.0, 75.2)	75.2

### Experiments on KIMIA24

In the second experiment, we used VGG on the KIMIA24 dataset with the deep features extracted by VGG on GTEx; the task is the identification of the slide of origin (24 classes). In the DAPPER framework, classifiers were trained on 1, 060 annotated tiles and validated on 265 unseen ones.

### UMAP analysis

In order to perform an unsupervised exploration of the features extracted by the Feature Extractor module, we projected the deep features onto a bi-dimensional space by using the Uniform Manifold Approximation and Projection (UMAP) multidimensional projection method. This dimension reduction technique, which relies on topological descriptors, has proven competitive with state-of-the-art visualization algorithms such as t-SNE [57], preserving both global and local structure of the data [58, 59]. We used the *R* *umap* package with following parameters: n_neighbors = 40, min_dist = 0.01, n_components = 2, and Euclidean metric.

### Implementation

All the code of the DAPPER framework is written in *Python* (v3.6) and *R* (v3.4.4). In addition to the general scientific libraries for Python, the scripts for the creation and training of the networks are based on *PyTorch*; the backend networks are implemented in *torchvision*. The library for processing histological images (available at gitlab.fbk.eu/mpba-histology/histolib) is based on *OpenSlide* and *scikit-image*.

The computations were performed on Microsoft Azure Virtual Machines with 4 NVIDIA K80 GPUs, 24 Intel Xeon E5-2690 cores and 256 GB RAM.

## Results

**Fig 3:**
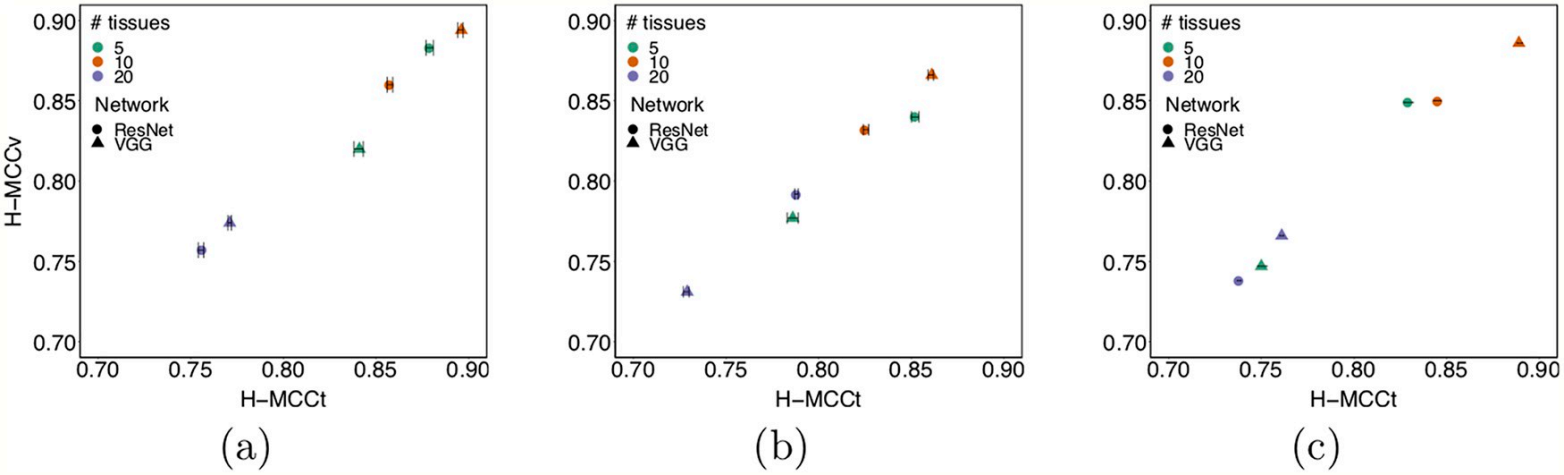
Comparison of DAPPER cross validation MCC (H-MCCt), vs MCC on external validation (H-MCCv) performance for each classifier. (a) FCH; (b) SVM; (c) RF.

Results of the tissue classification tasks in the DAPPER framework are listed in Table 4 for Matthews Correlation Coefficient (MCC) and Table 5 for Accuracy (ACC), respectively. See also Fig 3 for a comparison of MCC in internal cross validation with external validation.

All backend network-head pairs on HINT have MCC> 0.7 with narrow CIs, with estimates from internal validation close to performance on the external validation set (Fig 3). Agreement of internal estimates with values on external validation set is a good indicator of generalization and potential for reproducibility. All models reached their top MCC accuracy with 1, 000 features. On HINT5 and HINT10, the FCH neural network performs better than SVMs and RF. As expected, MCC ranged close to 0 for random labels; random ranking for increasing feature set sizes reached top MCC only for all features (tested for SVMs).

**Fig 4:**
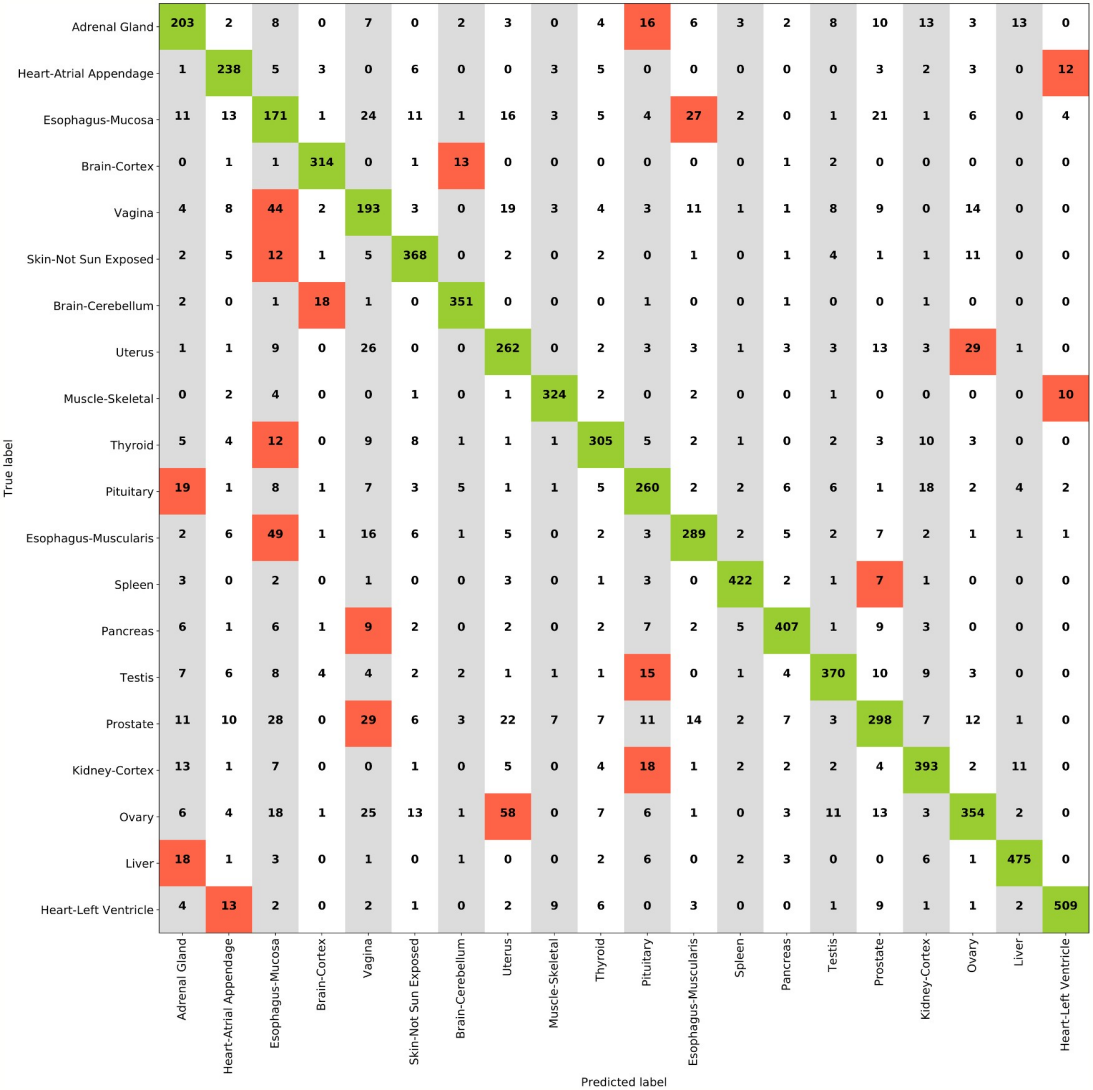
Confusion matrix for ResNet+SVM model on HINT20. Red shaded cells indicate the most confused classes.

The most accurate models both for internal and external validation estimates were the ResNet+FCH model with MCC = 0.883 on HINT5, the VGG+FCH model on HINT10, and the ResNet+SVM model on HINT20. In S6 Table we show the results with a lower number of dense layers in the FCH, which are comparable with the FCH with 4 dense layers. Results on HINT30 are detailed in S5 Table; on external validation set, the VGG model reaches accuracy ACC = 61.8% and MCC = 0.61. Performance decreases for more complex multiclass problems. Notably the difficulty of the task is also complicated by tissue classes that are likely to have similar histological patterns, such as misclassification of Esophagus-Muscularis (ACC: 72.1%) with Esophagus-Mucosa (ACC: 53.2%), or the two Heart tissue subtypes or the 58 Ovary(ACC: 68.3%) tiles predicted as Uterus (ACC: 72.8%). The full confusion matrix for ResNet with SVMs on HINT20 is reported in Fig 4. In this paper we establish a methodology to evaluate reproducibility and predictive accuracy of machine learning models, in particular of the model selection phase. This is obtained by moving from single training-test split procedure to an evaluation environment that uses data replicates and averaged statistical indicators, thus enabling to select a model on the basis of statistical indicators derived from the internal validation loop. In this framework, we can honestly evaluate model performance differences along a set of experiments on a group of tasks. The DAPPER framework cannot by itself identify the reason of such difference, and indeed the emergence of optimal architectures for a specific task may be due to different factors, as revealed by appropriate experimental design. In terms of the experimental design described in this paper, for any model type we expect and find a decrease in accuracy for increasing number of classes, which requires learning more decision surfaces with less data per class. Notably, the best model in the internal DAPPER validation is confirmed to be the best also on the unseen test data, with a value within the confidence interval or immediately close.

### Results on KIMIA24

Regardless of difference in image types, VGG-KIMIA24 with both RF and SVM heads with ACC = 43.4% (see Table 6), improving on published results (ACC = 41.8% [38]).

**Table 6:** Performance of DAPPER framework for VGG backend network, and classifier heads (FCH, SVM, RF) on KIMIA24 dataset. The average cross validation MCC (**K24-MCCt**), and ACC (**K24-ACCt**) with 95% CI, as well as MCC (**K24-MCCv**), and ACC (**K24-ACCv**) on external validation set are reported.

Model	K24-MCCt	K24-MCCv	K24-ACCt	K24-ACCv
VGG+FCH	0.317 (0.306, 0.327)	0.207	34.4 (33.2, 35.2)	23.8
VGG+SVM	0.446 (0.439, 0.454)	0.409	47.1 (46.4, 47.8)	43.4
VGG+RF	**0.457** (**0.449**, **0.465**)	**0.409**	**48.0** (**47.3**, **48.8**)	**43.4**

**Fig 5:**
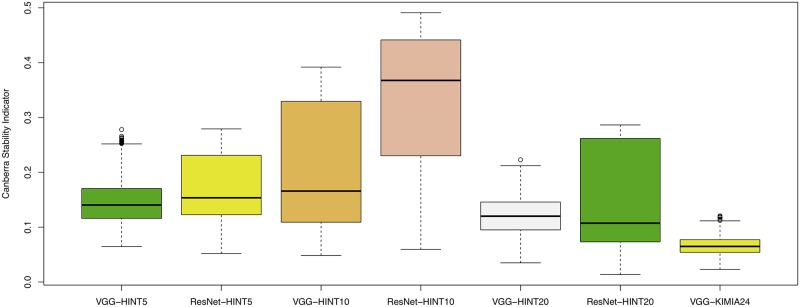
Canberra stability indicator on HINT and KIMIA datasets. For each architecture, a set of deep feature lists is generated, one list for each internal run of training in the nested cross validation schema, each ranked with KBest. Canberra stability is computed as in [32]: lower stability is better.

It is worth noting that transfer learning from ImageNet to HINT restricts training to the Adapter and Fully Connected Head blocks. In one-shot experiments, MCC further improves when the whole feature extraction block is retrained (see S3 Table). However, the result still needs to be consolidated by extending the DAP also to the training or retraining of the deep learning backend networks to check for actual generalization. The Canberra stability indicator was also computed for all the experiments, with minimal median stability for ResNet-20 (Fig 5).

### Results at WSI-level

We evaluated the performance of DAPPER at WSI-level on the HINT20 external validation set, with the ResNet+SVM model. In particular, all the predictions for the tiles are aggregated by WSI, and the resulting tissue will be the most common one among those predicted on the corresponding tiles. However, it is worth noting that the number of tiles per WSI in the HINT20 external validation set varies (min 1, max 31) due to a stratification strategy only considering the tissues-per-sample distribution (see Section *Data Analysis Plan*). Therefore, we restricted our evaluation to a subset of 15 WSI per class (300 WSI in total), each of which associated to 10 tiles randomly selected. This value represents a reasonable number of Regions of Interest (ROIs) a human pathologist would likely consider in his/her evaluations. In this regard, we further investigate how the DAPPER framework performs on an increasing number of tiles per WSI, namely 3, 5, 7, and 10. As expected, the overall accuracy improves as the number of tiles per WSI increases, reaching 98.3% when considering all 10 tiles per WSI. Notably, the accuracy is high even when reducing to 3 tiles per WSI (see Table 7).

**Table 7:** Metrics at WSI-level for increasing number of tiles per WSI. Metrics are computed on a subset of HINT20 external validation set, consisting of 15 WSI per class (300 WSI in total). The WSI class is determined by the most frequent predicted class by the *ResNet+SVM* model for the considered tiles.

# Tiles per WSI	MCC	ACC (%)
3	0.86	86.3
5	0.93	93.7
7	0.96	96.0
10	0.98	98.3

### Comparison with pathologist

**Fig 6:**
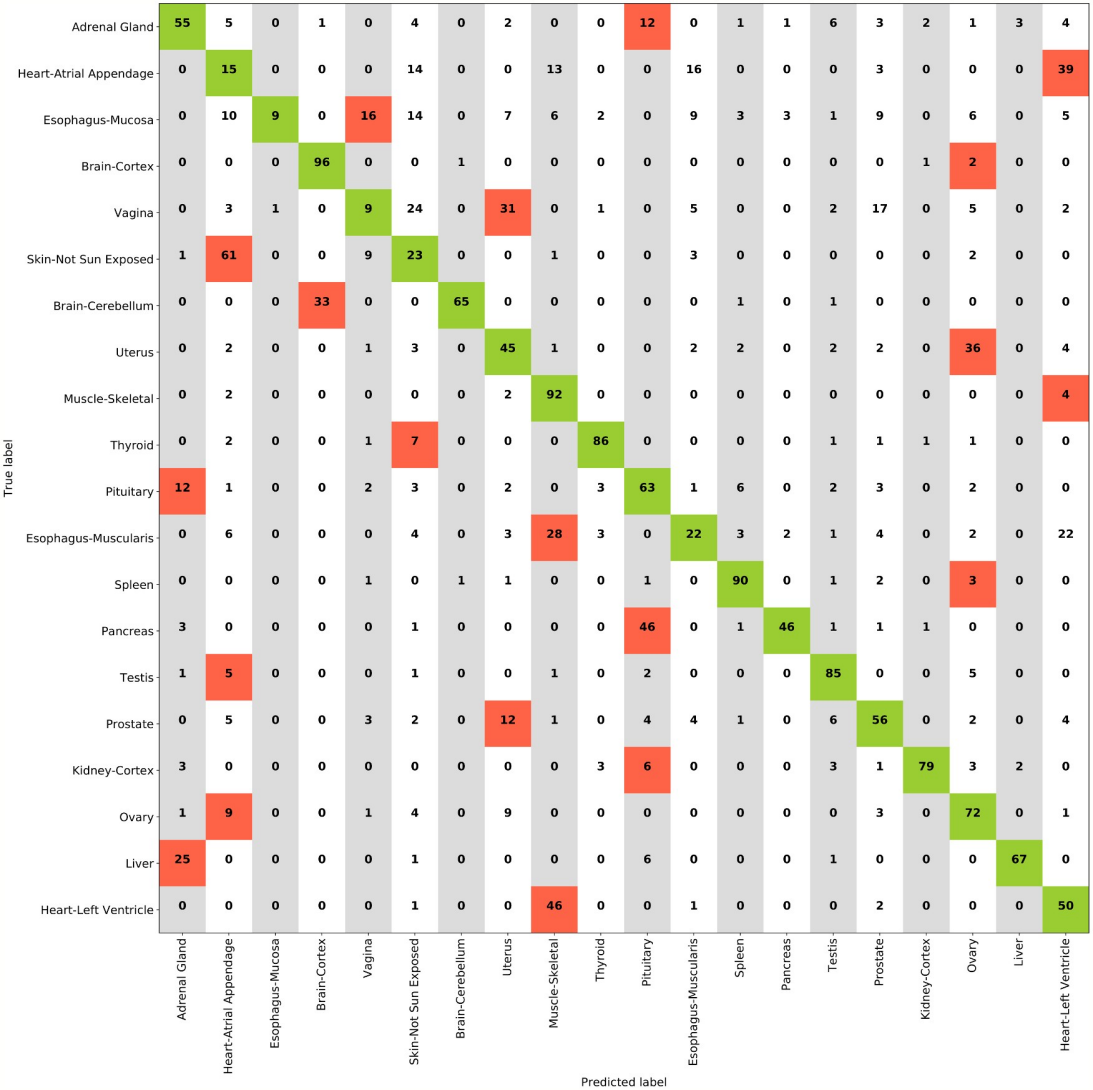
Confusion matrix for pathologist classification on a subset of HINT20 external validation set. Red shaded cells indicate the most confused classes.

We tested the performance of DAPPER against an expert pathologist on about 25% of the HINT20 external validation set, 2, 000 tiles out of 8, 103, with 100 randomly selected tiles for each class. We asked the pathologist to classify each tile by choosing among the 20 classes of the HINT20 dataset, without imposing any time constraint. The confusion matrix resulting from the evaluation of tiles as produced by the pathologist is shown in Fig 6. Predictions produced by the DAPPER framework for comparative results are then collected on the same data. The best-performing model on the HINT20 dataset, namely the ResNet+SVM model, has been considered for this experiment. As reported in Table 8, DAPPER outperforms the pathologist in the prediction of tissues at a tile-level.

**Table 8:** Tissue classification performance of DAPPER vs pathologist. DAPPER with *ResNet+SVM* model outperforms the pathologist at tile-level. Metrics are computed on a subset of HINT20 external validation set (2, 000 tiles).

Classifier	MCC	ACC (%)
Pathologist	0.542	56.3
DAPPER	0.786	79.6

To provide an unbiased estimation of the performance of DAPPER, we repeated the same evaluation on 10 other randomly generated subsets of 2, 000 tiles extracted from the HINT20 external validation set. The obtained average MCC and ACC with 95% CI are 0.786 (0.783, 0.789), and 79.6 (79.3, 79.9), respectively.

Finally, since the classification at tile-level is an unusual task for a pathologist, who is instead trained on examining the whole context of a tissue scan, as a second task we asked the pathologist to classify 200 randomly chosen WSIs (10 for each class of HINT20). As expected, the results in this case are better than those at tile-level, *i.e*., MCC = 0.788, and ACC = 79.5%, to be compared with the DAPPER performances reported in Table 7.

### The HINT benchmark dataset

As a second contribution of this study, we are making available the HINT dataset, generated by the first component of tools in the DAPPER framework, as a benchmark dataset for validating machine learning models in digital pathology. The HINT dataset is currently composed of 53, 727 tiles at 512 × 512 resolution, based on histology from GTEx. HINT can be easily expanded to over 78, 000 tiles, as for this study we used a fraction of the GTEx images and at most 100 tiles from each WSI were extracted. Digital pathology still misses a universally adopted dataset to compare deep learning models as already established in vision (*e.g*., ImageNet for image classification, COCO for image and instance segmentation). Several initiatives for a “BioImageNet” will eventually improve this scenario. Histology data are available in the generalist repository Image Data Resource (IDR) [60, 61]. Further, the International Immuno-Oncology Biomarker Working Group in Breast Cancer and the MAQC Society have launched a collaborative project to develop data resources and quality control schemes on Machine Learning algorithms to assess TILs in Breast Cancer.

HINT is conceptually similar to KIMIA24. However, HINT inherits from GTEx more variability in terms of sample characteristics, validation of donors and additional access to molecular data. Further, we used a random sampling approach to process tiles excluding background and minimize human intervention in the choice and preparation of the images.

### Deep features

**Fig 7:**
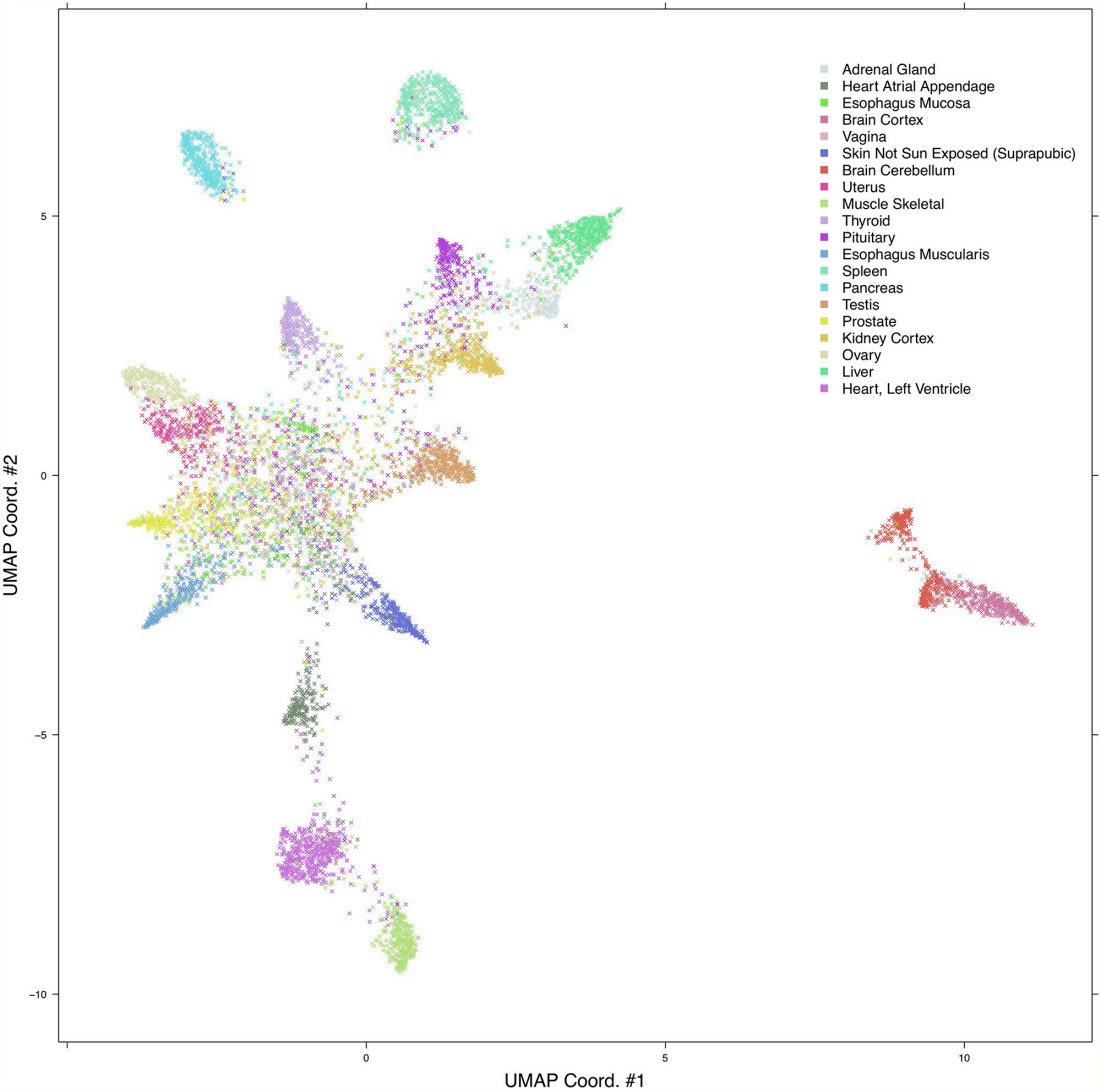
UMAP projection of external validation set for VGG-20 experiment.

We applied an unsupervised projection on all the features extracted by VGG and ResNet networks on all tissues tasks. In the following, we discuss an example for features extracted by VGG on the HINT20 task, displayed as UMAP projection (Fig 7), points are coloured for 20 tissue labels. The UMAP displays for the other tasks are available in S1–S4 Figs. The UMAP display is in agreement with the count distributions in the confusion matrix (Fig 4). The deep learning embedding separates well a set of histology types, including Muscle-Skeletal, Spleen, Pancreas, Brain-Cortex and Cerebellum, Heart-Left Ventricle and Atrial Appendage which group into distinct clusters (See Fig 7 and Table 9). The distributions of the activations for the top-3 deep features of the VGG backend network on the HINT10 dataset are displayed in S5 Fig; the top ranked deep feature (#668) is clearly selective for Spleen. The UMAP projection also shows an overlapping for tissues such as Ovary and Uterus, or Vagina and Esophagus-Mucosa, or the two Esophagus histotypes, consistently with the confusion matrix (Fig 4).

**Table 9:** Histology types well separated by SVM+ResNet model for HINT20. Accuracy is computed with respect to the confusion matrix in Fig 4 and expressed in percentage, together with the total number of samples for each class.

Histology type	ACC(%)	#samples
Spleen	94.6	446
Brain—Cortex	94.3	333
Muscle—Skeletal	93.4	347
Brain—Cerebellum	93.4	376
Heart—Left Ventricle	90.1	565
Pancreas	87.9	463
Heart—Atrial Appendage	84.7	317

**Fig 8:**
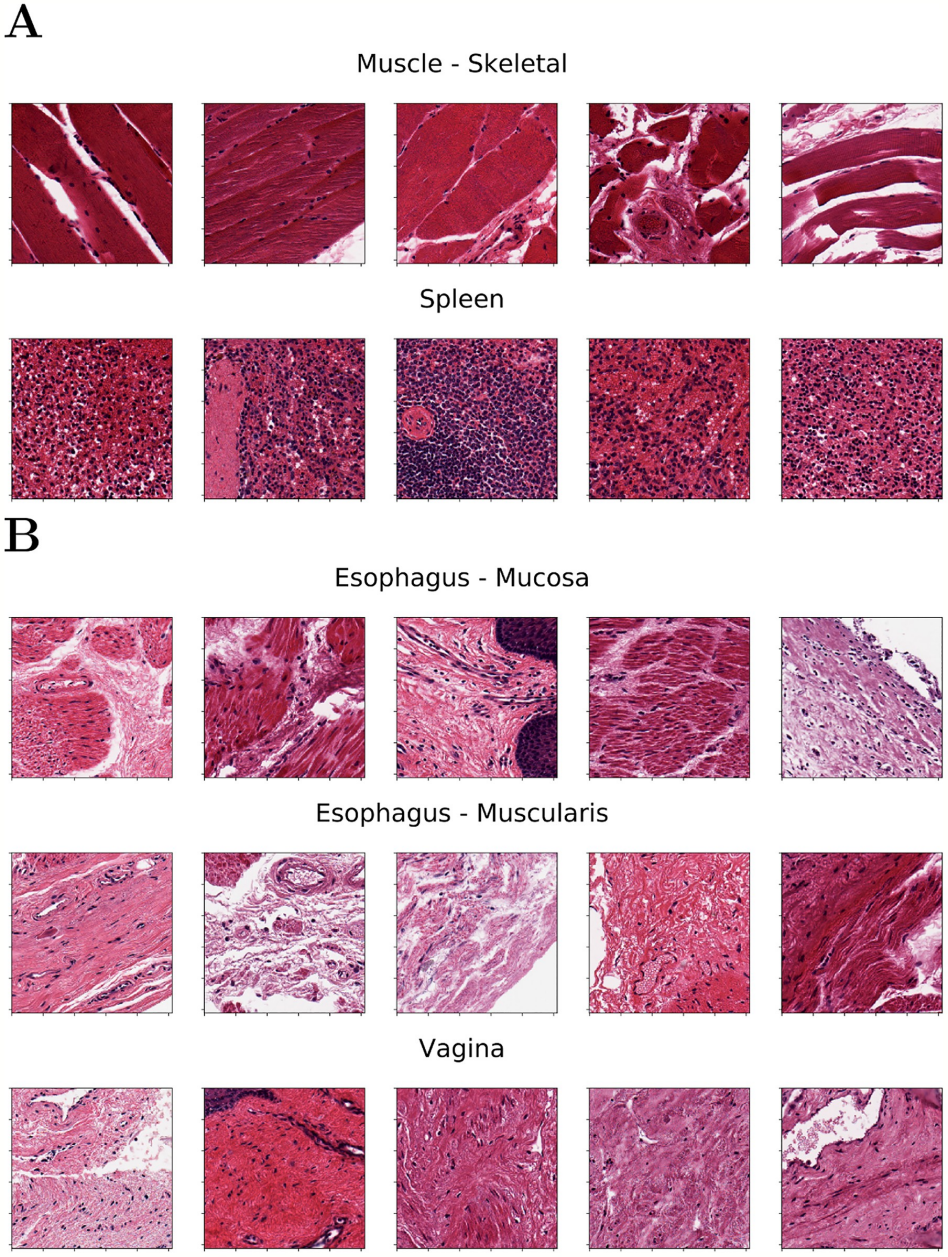
Representative tiles predicted from VGG-20 experiment. A) Examples from two well-separated clusters observed in the UMAP embedding. B) Samples of mislabeled tiles from tissues partially overlapping in the UMAP embedding.

Examples of five tiles from two well separated clusters, Muscle-Skeletal (ACC: 93.4%) and Spleen (ACC: 94.6%), are displayed in panel A of Fig 8. Tiles from three clusters partially overlapping in the neural embedding and mislabeled in both the VGG-20 and ResNet-20 embeddings with SVM (Esophagus- Mucosa ACC = 53.2%, Esophagus-Muscularis ACC = 72.1%, Vagina ACC = 59.0%) are similarly visualized in Fig 8B. While the aim of this paper is to introduce a framework for honest comparison of models that will be used for clinical purposes rather than fine-tuning accuracy in this experiment, it is evident that these tiles have morphologies that are hard to classify. This challenge requires more complex models (*e.g*. ensembles) and a structured output labeling, already applied in dermatology [2]. Further, we are exploring the combination of DAPPER with image analysis packages, such as HistomicsTK (https://digitalslidearchive.github.io/HistomicsTK/) or CellProfiler [62], to extract features useful for interpretation and feedback from pathologists.

## Discussion

Digital pathology would greatly benefit from the adoption of machine learning, shifting human assessment of histology to higher quality, non-repetitive tasks. Unfortunately, there is no fast, easy route to improve reproducibility of automated analysis. The adoption of the DAP clearly sets in a computational aggravation not usually considered for image processing exercises. However, this is an established practice with massive omics data [28], and reproducibility by design can handle secondary results useful for diagnostics and for interpretation.

We designed the DAPPER framework as a tool for evaluating accuracy and stability of deep learning models, currently only backend elements in a sequence of processing steps, and possibly in the future end-to-end solutions. We choose as test domain H&E stained WSIs for prediction of tissue of origin, which is not a primary task for trained pathologists, but a reasonable benchmark for machine learning methods. Also, we are aware that tissue classification is only a step in real digital pathology applications. Mobadersany and colleagues [11] used a deep learning classifier to score and visualize risk on the WSIs. Similarly, deep learning tile classification may be applied to quantify histological differences in association to a genomic pattern, *e.g*., a specific mutation or a high-dimensional protein expression signature. In this vision, the attention to model selection supported by our framework is a prerequisite for developing novel AI algorithms for digital pathology, *e.g*., for analytics over TILs.

Although we are building on deep learning architectures known for applications on generic images, they adapted well to WSIs in combination with established machine learning models (SVM, RF); we expect that large scale bioimaging resources will give the chance of improving the characterization of deep features, as already emerged with the HINT dataset that we are providing as public resource. In this direction, we plan to release the network weights of the backend DAPPER models that are optimized for histopathology as alternative pretrained weights for digital pathology, similarly to those for the ImageNet dataset and available in torchvision.

## Supporting information

S1 Table Summary of available samples, downloaded WSIs and extracted tiles. (PDF)

S2 Table Summary of the datasets. (PDF)

S3 Table Impact of retraining the backend network. Accuracy and Matthews Correlation Coefficient improve when retraining also the feature extraction block (VGG backend network, not in DAP). We observe an improvement of the accuracy from 5.5% to 24.8% for the four chosen experiments. Possibly the neural network benefits from adjusting also the initial weights because the layers learn characteristics of the images diverse from the ImageNet dataset.
(PDF)

S4 TableComparison of the three optimization methods to set the learning rate.The best method for setting the learning rate was assessed using the VGG as backend network on the 5 tissues dataset HINT5. Three methods were tested: Fixed (FIX): the learning rate is set to 10^−5^ for the whole training; Step-wise (STEP): the learning rate is initialized at λ_init_ = 10^−3^ and updated every 10 epochs with the following rule: λ_new_ = λ_old_/10; Polynomial (POLY): the learning rate is initialized at 10^−3^ and updated every 10 iterations with a polynomial law: $\lambda_{\text{new}}=\lambda_{\text{init}}{\left(1-\frac{i}{I_{\text{max}}}\right)}^{0.9}$, where *i* is the index of the iteration and *I*_*max*_ is the total number of iterations.(PDF)

S5 Table Impact of task complexity (VGG backend network). Performance decreases when the number of tissues increases. Adding more classes to the task is possibly complicated by the introduction of tissues with similar histological patterns.
(PDF)

S6 Table Impact (MCC) of number of internal layers on FCH (< 4 dense layers) on HINT dataset. FCH3: three dense layers with 1000, 256 and # tissue classes nodes, respectively; FCH2: two dense layers with 256 and # tissue classes nodes, respectively. The average cross validation MCC with 95% CI (H-MCCt), and MCC on the external validation set (H-MCCv) are reported. In bold: MCC (bold) values of Table 4 of the main text.
(PDF)

S1 Fig UMAP projection on training (circles) and external validation (crosses) set for VGG-5 experiment. (PNG)

S2 Fig UMAP projection on training (circles) and external validation (crosses) set for ResNet-5 experiment. (PNG)

S3 Fig UMAP projection on training (circles) and external validation (crosses) set for VGG-10 experiment. (PNG)

S4 Fig UMAP projection on training (circles) and external validation (crosses) set for ResNet-10 experiment. (PNG)

S5 Fig Deep features and tissue of origin. Distributions of the values of the top-3 deep features computed with the VGG backend architecture for the 10 classes of the HINT10 dataset.
(PDF)
