# Supplementary material for: Evaluating reproducibility of AI algorithms in digital pathology with DAPPER
Source: PLoS Comput Biol. 2019 Mar 27;15(3):e1006269. doi: 10.1371/journal.pcbi.1006269 (PMC6467397; doi:10.1371/journal.pcbi.1006269)
Supplement: S2 Table — (PDF) [file pcbi.1006269.s002.pdf]

Dataset	Anatomical zone [# Tiles samples]
HINT5	Kidney - Cortex [2424], Pancreas [2193], Colon - Transverse [1301], Breast - Mammary Tissue [1291], Lung [1009].
HINT10	Heart - Left Ventricle [2689], Liver [2583], Ovary [2452], Kidney - Cortex [2424], Prostate [2323], Testis [2221], Pancreas [2193], Spleen [2184], Esophagus - Muscularis [1926], Pituitary [1890].
HINT20	Heart - Left Ventricle [2689], Liver [2583], Ovary [2452], Kidney - Cortex [2424], Prostate [2323], Testis [2221], Pancreas [2193], Spleen [2184], Esophagus - Muscularis [1926], Pituitary [1890], Thyroid [1890], Muscle - Skeletal [1875], Uterus [1857], Brain - Cerebellum [1825], Skin - Not Sun Exposed (Suprapubic) [1811], Vagina [1746], Brain - Cortex [1731], Esophagus - Mucosa [1700], Heart - Atrial Appendage [1622], Adrenal Gland [1574].
HINT30	Heart - Left Ventricle [2689], Liver [2583], Ovary [2452], Kidney - Cortex [2424], Prostate [2323], Testis [2221], Pancreas [2193], Spleen [2184], Esophagus - Muscularis [1926], Pituitary [1890], Thyroid [1890], Muscle - Skeletal [1875], Uterus [1857], Brain - Cerebellum [1825], Skin - Not Sun Exposed (Suprapubic) [1811], Vagina [1746], Brain - Cortex [1731], Esophagus - Mucosa [1700], Heart - Atrial Appendage [1622], Adrenal Gland [1574], Stomach [1477], Esophagus - Gastroesophageal Junction [1440], Colon - Sigmoid [1401], Colon - Transverse [1301], Breast - Mammary Tissue [1291], Nerve - Tibial [1286], Small Intestine- Terminal Ileum [1184], Skin - Sun Exposed (Lower leg) [1129], Lung [1009], Artery - Tibial [957].
