# Supplementary material for: Evaluating reproducibility of AI algorithms in digital pathology with DAPPER
Source: PLoS Comput Biol. 2019 Mar 27;15(3):e1006269. doi: 10.1371/journal.pcbi.1006269 (PMC6467397; doi:10.1371/journal.pcbi.1006269)
Supplement: S3 Table — Accuracy and Matthews Correlation Coefficient improve when retraining also the feature extraction block (VGG backend network, not in DAP). We observe an improvement of the accuracy from 5.5% to 24.8% for the four chosen experiments. Possibly the neural network benefits from adjusting also the initial weights because the layers learn characteristics of the images diverse from the ImageNet dataset. (PDF) [file pcbi.1006269.s003.pdf]

	FCH			
	VGG retrained		VGG not retrained	
Experiment	ACC %	MCC	ACC %	MCC
VGG-5	97.6	0.970	92.1	0.901
VGG-10	97.5	0.972	88.6	0.874
VGG-20	93.9	0.936	76.9	0.759
VGG-30	77.1	0.765	61.8	0.607
