# Supplementary material for: Evaluating reproducibility of AI algorithms in digital pathology with DAPPER
Source: PLoS Comput Biol. 2019 Mar 27;15(3):e1006269. doi: 10.1371/journal.pcbi.1006269 (PMC6467397; doi:10.1371/journal.pcbi.1006269)
Supplement: S4 Table — The best method for setting the learning rate was assessed using the VGG as backend network on the 5 tissues dataset HINT5. Three methods were tested: Fixed (FIX): the learning rate is set to 10−5 for the whole training; Step-wise (STEP): the learning rate is initialized at λinit = 10−3 and updated every 10 epochs with the following rule: λnew = λold/10; Polynomial (POLY): the learning rate is initialized at 10−3 and updated every 10 iterations with a polynomial law: λnew=λinit(1-iImax)0.9, where i is the index of the iteration and Imax is the total number of iterations. (PDF) [file pcbi.1006269.s004.pdf]

	FCH	
Opt. method	ACC	MCC
**FIX**	**93.7**	**0.921**
STEP	88.4	0.854
POLY	91.1	0.888
