# Supplementary material for: Evaluating reproducibility of AI algorithms in digital pathology with DAPPER
Source: PLoS Comput Biol. 2019 Mar 27;15(3):e1006269. doi: 10.1371/journal.pcbi.1006269 (PMC6467397; doi:10.1371/journal.pcbi.1006269)
Supplement: S5 Table — Performance decreases when the number of tissues increases. Adding more classes to the task is possibly complicated by the introduction of tissues with similar histological patterns. (PDF) [file pcbi.1006269.s005.pdf]

	FCH	
Experiment	ACC %	MCC
VGG-5	93.7	0.921
VGG-10	88.6	0.874
VGG-20	76.9	0.760
VGG-30	61.8	0.607
