# Supplementary material for: Evaluating reproducibility of AI algorithms in digital pathology with DAPPER
Source: PLoS Comput Biol. 2019 Mar 27;15(3):e1006269. doi: 10.1371/journal.pcbi.1006269 (PMC6467397; doi:10.1371/journal.pcbi.1006269)
Supplement: S6 Table — FCH3: three dense layers with 1000, 256 and # tissue classes nodes, respectively; FCH2: two dense layers with 256 and # tissue classes nodes, respectively. The average cross validation MCC with 95% CI (H-MCCt), and MCC on the external validation set (H-MCCv) are reported. In bold: MCC (bold) values of Table 4 of the main text. (PDF) [file pcbi.1006269.s006.pdf]

	FCH3		FCH2	
Experiment	H-MCCt	H-MCCv	H-MCCt	H-MCCv
VGG-5	0.841 (0.838, 0.843)	0.823	0.832 (0.829, 0.834)	0.822
ResNet-5	**0.881 (0.878, 0.883)**	**0.887**	0.871 (0.869, 0.873)	0.877
VGG-10	**0.895 (0.894, 0.896)**	**0.895**	0.893 (0.892, 0.895)	0.892
ResNet-10	0.858 (0.856, 0.859)	0.860	0.853 (0.851, 0.854)	0.855
VGG-20	0.773 (0.772, 0.774)	0.775	0.768 (0.766, 0.769)	0.769
ResNet-20	0.757 (0.756, 0.759)	0.756	0.749 (0.748, 0.751)	0.746
