# Supplementary figures and images for: Evaluating reproducibility of AI algorithms in digital pathology with DAPPER
Source: PLoS Comput Biol. 2019 Mar 27;15(3):e1006269. doi: 10.1371/journal.pcbi.1006269 (PMC6467397; doi:10.1371/journal.pcbi.1006269)

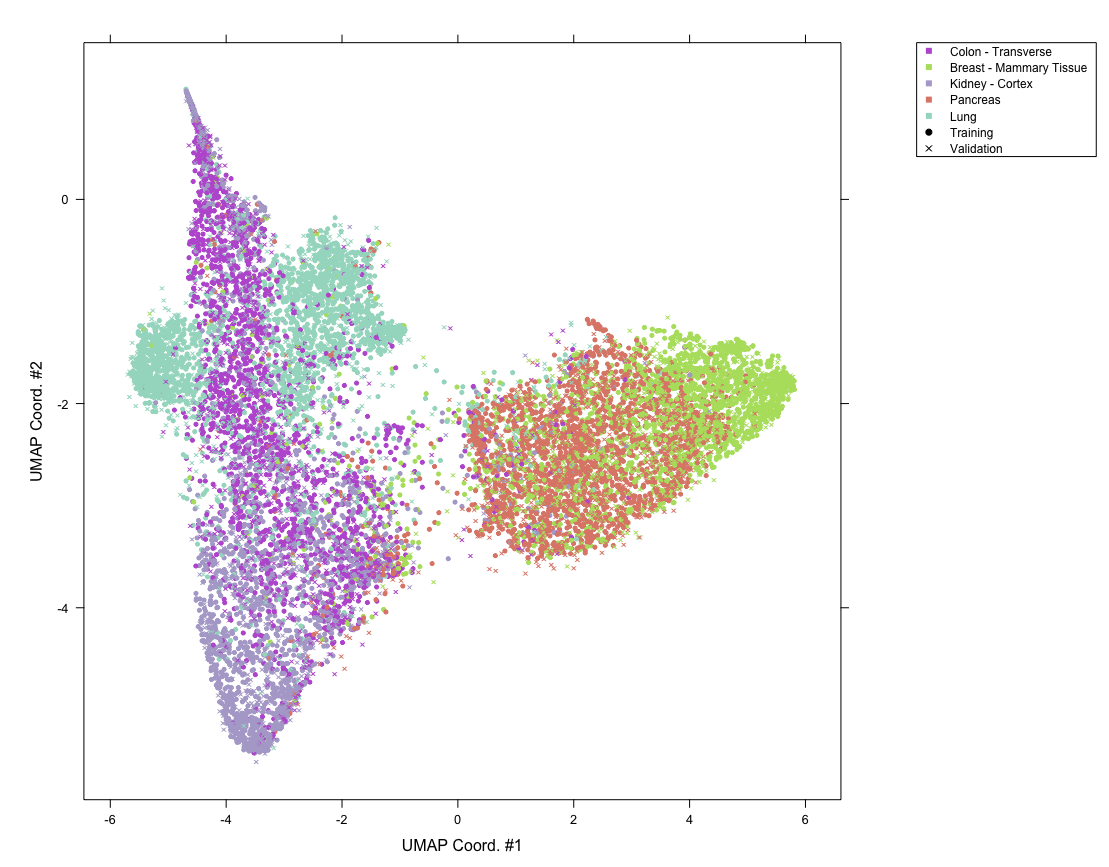

Supplement: S1 Fig — (PNG) [file pcbi.1006269.s007.png]

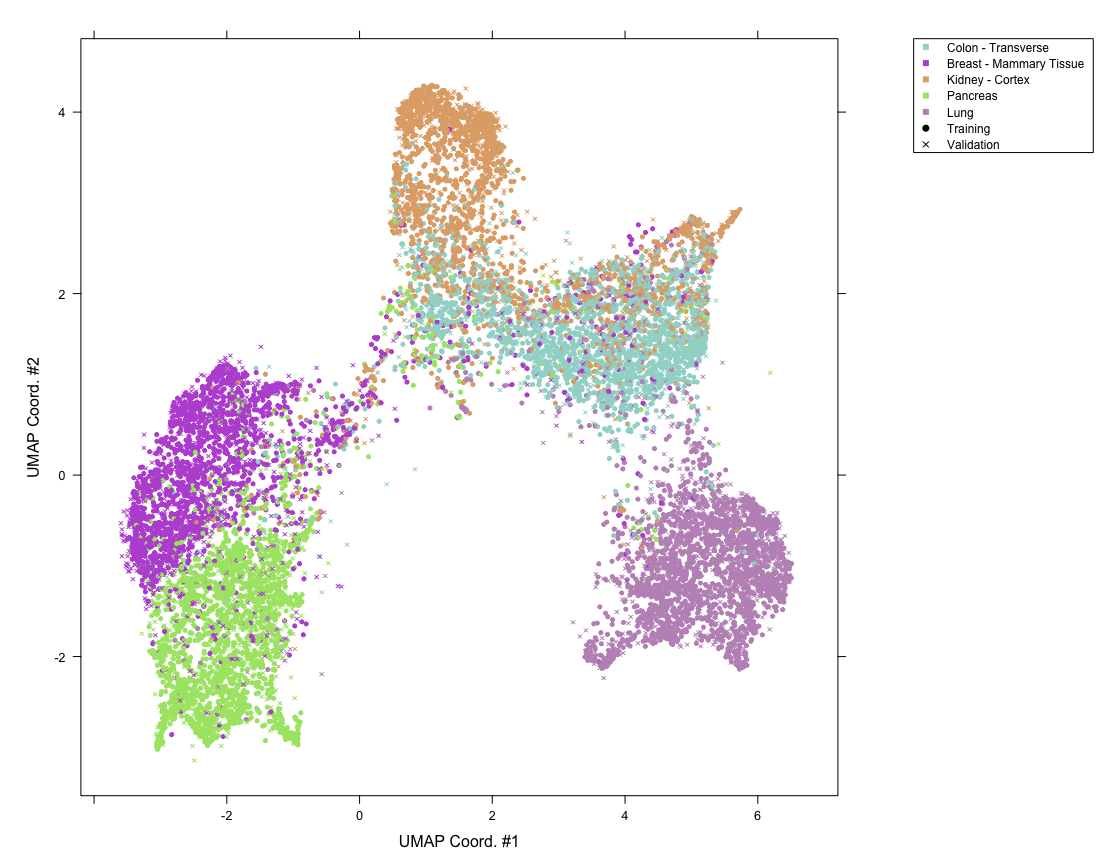

Supplement: S2 Fig — (PNG) [file pcbi.1006269.s008.png]

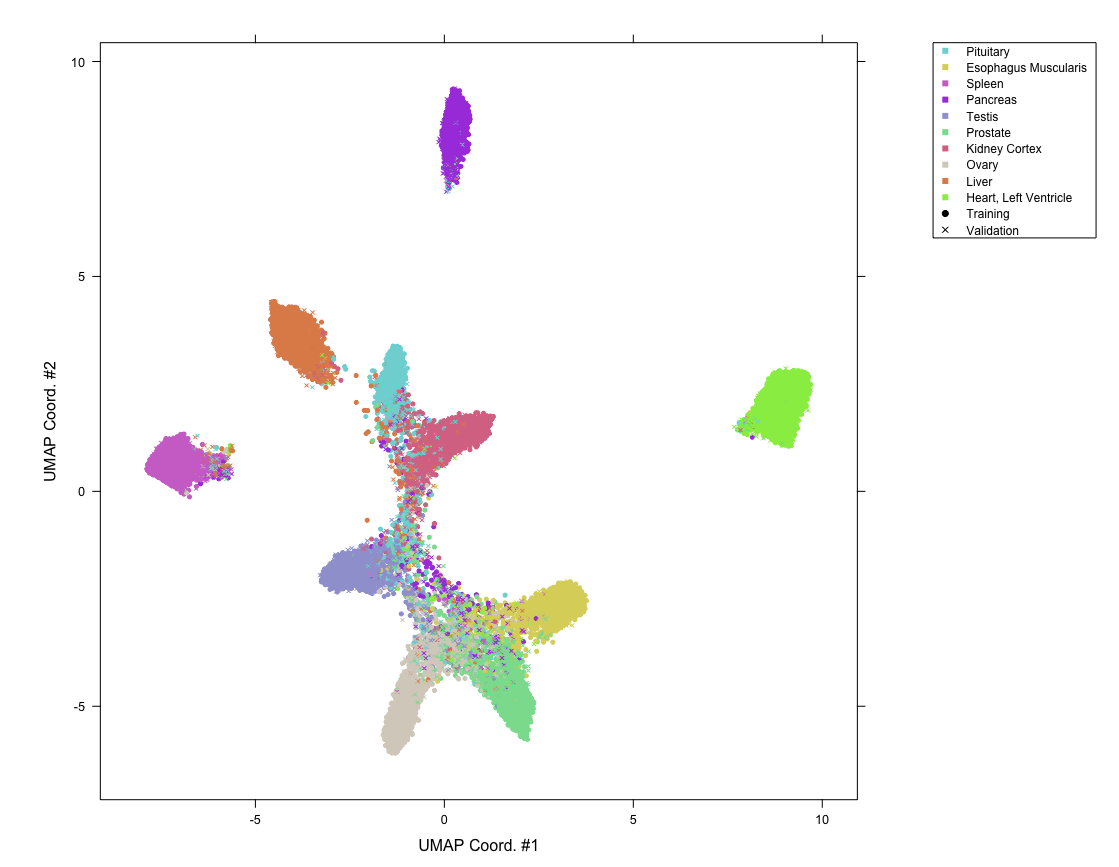

Supplement: S3 Fig — (PNG) [file pcbi.1006269.s009.png]

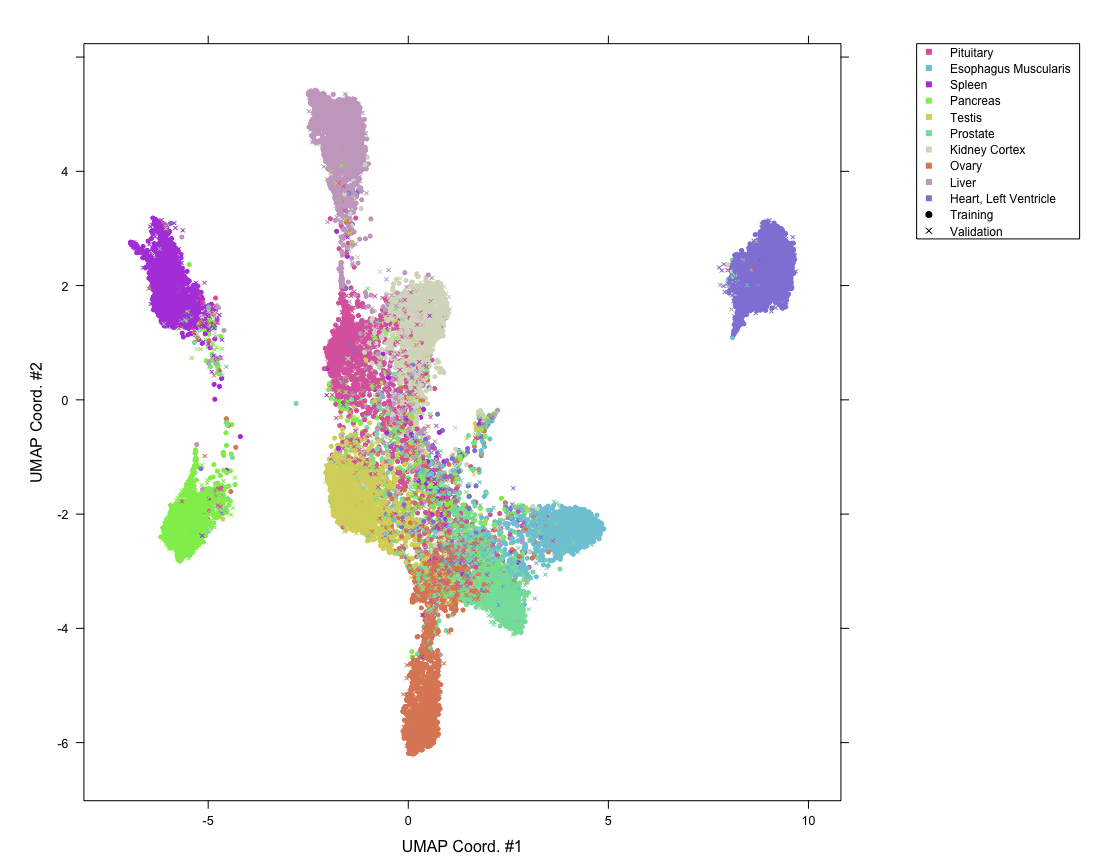

Supplement: S4 Fig — (PNG) [file pcbi.1006269.s010.png]

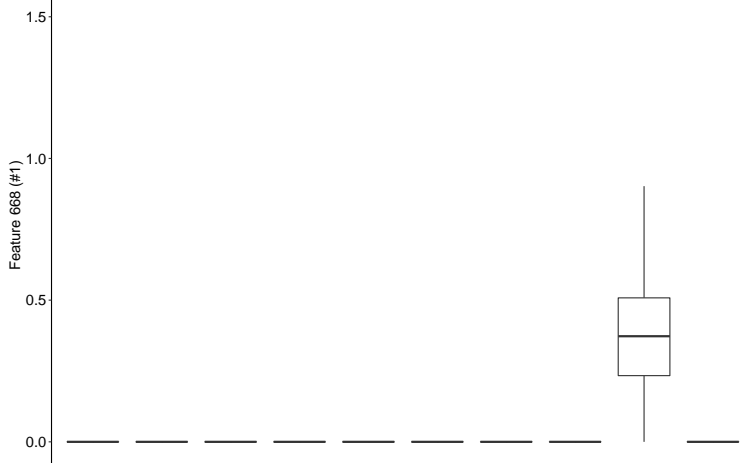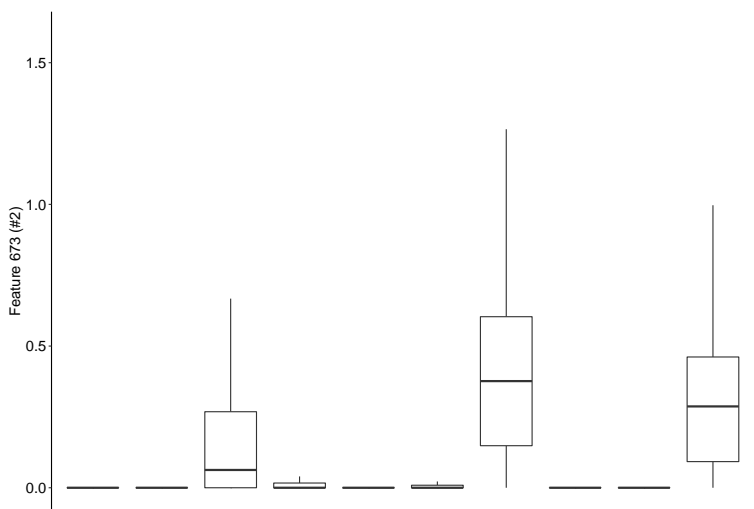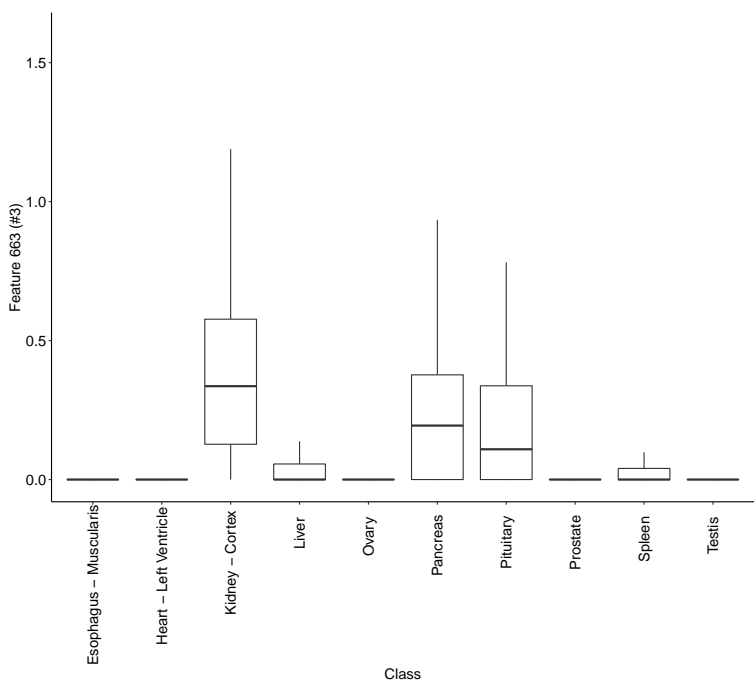

Supplement: S5 Fig — Distributions of the values of the top-3 deep features computed with the VGG backend architecture for the 10 classes of the HINT10 dataset. (PDF) [file pcbi.1006269.s011.pdf]
