# Supplementary material for: Evaluating reproducibility of AI algorithms in digital pathology with DAPPER
Source: PLoS Comput Biol. 2019 Mar 27;15(3):e1006269. doi: 10.1371/journal.pcbi.1006269 (PMC6467397; doi:10.1371/journal.pcbi.1006269)
Supplement: S1 Table — (PDF) [file pcbi.1006269.s001.pdf]

Anatomical part	# WSIs	# Tiles
1. Adipose - Subcutaneous	18	174
2. Adipose - Visceral (Omentum)	21	59
3. Adrenal Gland	25	1574
4. Artery - Aorta	19	876
5. Artery - Coronary	23	915
6. Artery - Tibial	22	957
7. Bladder	-	-
8. Brain - Cerebellum	20	1825
9. Brain - Cortex	19	1731
10. Breast - Mammary Tissue	26	1291
11. Cells - EBV-transformed lymphocytes	-	-
12. Cells - Transformed fibroblasts	-	-
13. Cervix - Ectocervix	-	-
14. Cervix - Endocervix	-	-
15. Colon - Sigmoid	21	1401
16. Colon - Transverse	20	1301
17. Esophagus - Gastroesophageal Junction	22	1440
18. Esophagus - Mucosa	23	1700
19. Esophagus - Muscularis	25	1926
20. Fallopian Tube	-	-
21. Heart - Atrial Appendage	20	1622
22. Heart - Left Ventricle	28	2689
23. Kidney - Cortex	28	2424
24. Liver	26	2583
25. Lung	21	1009
26. Minor Salivary Gland	19	896
27. Muscle - Skeletal	23	1875
28. Nerve - Tibial	23	1286
29. Ovary	26	2452
30. Pancreas	26	2193
32. Pituitary	21	1890
32. Prostate	24	2323
33. Skin - Not Sun Exposed (Suprapubic)	25	1811
34. Skin - Sun Exposed (Lower leg)	22	1129
35. Small Intestine - Terminal Ileum	24	1184
36. Spleen	22	2184
37. Stomach	21	1477
38. Testis	26	2221
39. Thyroid	26	1890
40. Uterus	21	1857
41. Vagina	21	1746
42. Whole Blood	-	-
